# Beyond the Needle: Reimagining Insulin Delivery and the Evolution of Inhaled Insulins

**DOI:** 10.7759/cureus.102856

**Published:** 2026-02-02

**Authors:** Prasanna Kumar K.M., Vijay Viswanathan, Banshi Saboo, Debmalya Sanyal, Kalyan K Gangopadhyay, Tirthankar Chaudhuri, Vageesh S Ayyar, Manoj Chawla, Ameya Joshi, Amit Gupta, Amarnath Sugumaran, Senthilnathan Mohanasundaram, Jaideep Gogtay, Supratik Bhattacharyya

**Affiliations:** 1 Department of Endocrinology, Center for Diabetes and Endocrine Care (CDEC), Bengaluru, IND; 2 Department of Diabetes and Endocrinology, M. Viswanathan (MV) Hospital for Diabetes, Chennai, IND; 3 Department of Diabetes and Endocrinology, Prof. M. Viswanathan Diabetes Research Centre, Chennai, IND; 4 Department of Diabetes, Diabetes Care and Hormone Clinic, Ahmedabad, IND; 5 Department of Endocrinology, KPC Medical College, Kolkata, IND; 6 Department of Diabetology, CK Birla Hospitals, Kolkata, IND; 7 Department of Diabetes and Endocrinology, Apollo Gleneagles Hospital, Kolkata, IND; 8 Department of Endocrinology, St. John’s Medical College and Hospital, Bengaluru, IND; 9 Department of Diabetes and Endocrinology, Lina Diabetes Care Centre, Mumbai, IND; 10 Department of Endocrinology, Bhaktivedanta Hospital and Research Institute, Thane, IND; 11 Department of Diabetes and Endocrinology, Centre for Diabetes Care, Greater Noida, IND; 12 Department of Medical Affairs, Cipla Ltd., Mumbai, IND; 13 Department of Endocrinology, SKN Diabetes and Endocrine Centre, Kolkata, IND

**Keywords:** barriers, inhaled insulin, injectable insulin, new insulin routes, subcutaneous insulin

## Abstract

Insulin has been around for a century since its discovery and has seen multiple innovations to improve its pharmacokinetic profile, thus impacting its efficacy, safety, and patient compliance. Despite its powerful glycemic efficacy, the use of insulin has remained sub-optimal owing mainly to the invasive route of administration and associated barriers. Non-invasive insulin development has been one of the highly researched fields in diabetes management. Amongst the various non-invasive routes investigated for insulin delivery, the pulmonary route has been among the most evaluated ones - a route that has yielded the only existing non-invasive option, inhaled insulin. Technosphere® insulin (Afrezza®; Mannkind Corporation, Danbury, CT, USA) is the only available inhaled insulin that has regulatory approval for the treatment of adult individuals with diabetes mellitus and has been available for more than a decade.

This review article provides an overview of the pulmonary route of administration and charts the developmental journey of inhaled insulins, with a focus on Technosphere® insulin.

## Introduction and background

Diabetes prevalence and burden have reached alarming proportions globally, and in India as well. The International Diabetes Federation (IDF) projects that by 2050, approximately 853 million adults will live with diabetes, up by 45% from that in 2024, i.e., 589 million [1]. The Indian Council of Medical Research-India Diabetes (ICMR-INDIAB) study estimated that approximately 101 million adult people with diabetes (PwD) live in India [2].

Insulin is the mainstay of treatment in type 1 diabetes mellitus (T1DM), and patients with type 2 diabetes mellitus (T2DM) often require insulin therapy when hyperglycemia is not controlled with other antihyperglycemic drugs. Insulin was discovered in 1921, and over the last 100 years, many advances have been made to deliver safer and more effective preparations [3].

**Figure 1:**
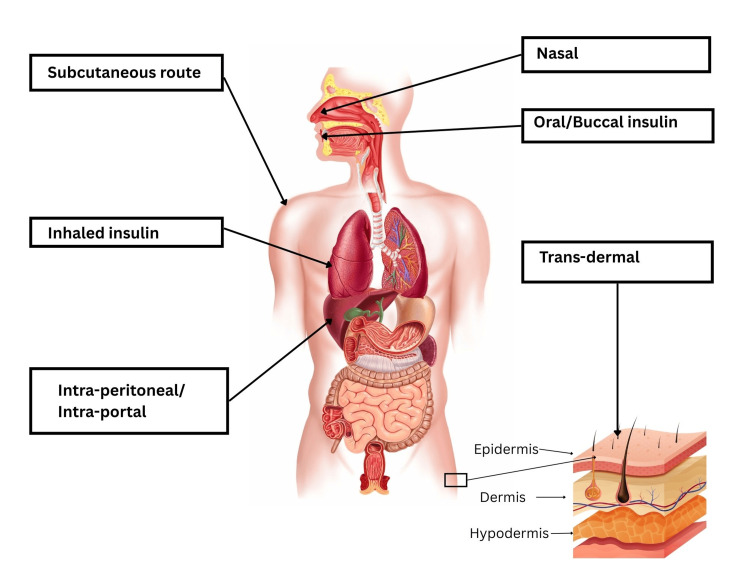
Routes of evaluated insulin administration Credit: [11]

Insulin is primarily administered subcutaneously. Several barriers hinder the initiation and intensification of injectable insulin therapy, occurring at the levels of PwD, healthcare providers, and the healthcare system [4-6]. Of these, the PwD level barriers are important as they affect treatment acceptance and adherence. Many PwDs do not easily accept this injectable route of administration because of several reasons, with the key ones being - the requirement of daily and often multiple injections, fear of needles, and social stigma associated with taking injections in public places [3-7]. Further, though insulin is a potent glucose-lowering drug, it is associated with certain risks such as hypoglycemia and weight gain [8]. The fear of hypoglycemia and weight gain is also a key barrier to insulin uptake [7]. Once started, treatment adherence is an issue with injectable insulin. To avoid the associated barriers of injectable insulin therapy, several alternative non-injectable routes of administration, such as oral, buccal, rectal, transdermal, and pulmonary, have been investigated (Figure 1) [9,10].

Of the various routes evaluated, inhaled or pulmonary insulin administration has been the only one approved by the United States Food and Drug Administration (US FDA) for use in PwD. Thus, it raises the possibility of improving treatment adherence by providing a non-injectable, easy-to-use route of insulin administration [3,8].

The first inhaled insulin was approved almost two decades ago, and since then, there have been developments in the field of insulin therapy. The Technosphere® insulin (Afrezza®; Mannkind Corporation, Danbury, CT, USA) was approved in 2014 and continues to be available in the US post approval [3,12]. However, its uptake in the US has been modest in insulin-treated patients, largely due to pulmonary monitoring requirements, higher cost, and limited insurance coverage [13-17].

This article provides a review of evidence available regarding the pulmonary route of administration for insulin, highlighting the advantages, efficacy, safety, challenges, and future potential associated with this novel route of insulin delivery. In addition, the review discusses the specific benefits of Afrezza® that may support broader clinical adoption and improve its overall uptake.

## Review

Relevant literature was identified through searches of major scientific databases, including PubMed and Google Scholar, using keywords such as inhaled insulin, Technosphere® insulin, pulmonary insulin, and Afrezza®. The review included peer-reviewed articles, clinical studies, and regulatory documents published in English. Additional sources were identified by screening reference lists of key publications.

The collected evidence was organized according to major thematic areas: the rationale for pulmonary insulin delivery, the physiology of inhaled insulin, methods used to deliver inhaled insulin, the historical development of inhaled insulin, and the characteristics of Technosphere® insulin. The data was organized to describe technological advancements, clinical performance, safety considerations, limitations, and future directions. This narrative approach enabled the integration of diverse information sources without performing a formal systematic quality assessment.

Pulmonary route

Novel Non-invasive Route for Insulin Administration

The deep lung tissue offers an excellent route for the administration of insulin owing to multiple factors, as shown in Table 1 [3,9,18,19]. It provides a very large surface area for drug absorption (50-140 m²) and aerates roughly 500 million alveoli with each breath. The alveolar epithelial membrane is extremely thin, and the alveolar-capillary network is dense, supporting efficient transcytosis and solute exchange. Alveoli are highly permeable to many biologics, enabling relatively rapid, first-order absorption.

**Table 1:** Advantages and suitability of the pulmonary route for insulin administration Source: [3,9,18,19]

Advantages and suitability of the pulmonary route for insulin administration
Enormous surface area (50-140 m²)
Aeration of roughly 500 million alveoli with each breath
Ultra-thin alveolar epithelial membrane
Extensive alveolar-capillary network for transcytosis and solute exchange ability
High permeability of alveoli to many biologics
Relatively rapid, first-order absorption
Less first-pass metabolism and degradation in the liver and gastrointestinal tract

Further, there is an inverse relationship between the molecular mass of a molecule and its absorption through the alveolar-capillary interface. Hence, the pulmonary route is suitable for administering peptides like insulin (around 6000 Da). For delivery to the lung alveoli (site of drug absorption), particles with a small size, i.e., < 3 μm, are required [20].

These factors facilitate the rapid absorption of proteins such as insulin into the systemic circulation. The use of the pulmonary route for the delivery of insulin was first reported as early as 1925 [21]. However, it’s only the advent of the 21st century that saw products reaching the clinical stage of development. Among all the inhaled insulin formulations being developed in the last 25 years, only two have received regulatory approval - Exubera® (Pfizer Inc., New York City, NY, USA) [22] and Afrezza® (Mannkind Corporation) [3,12]. Currently, Afrezza® is the only inhaled insulin approved and marketed anywhere in the world. 

Physiology of Inhaled Insulin

**Figure 2:**
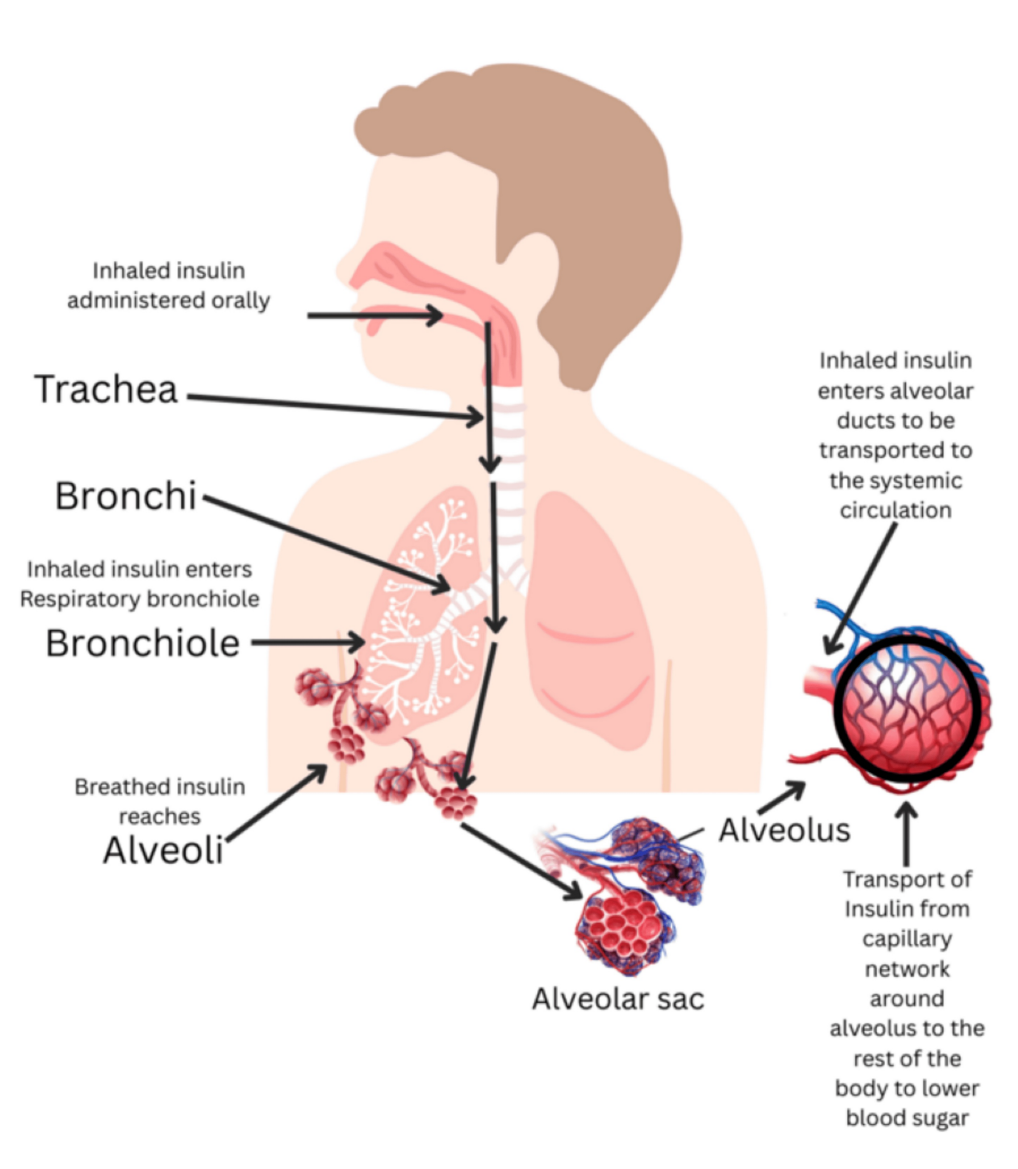
Route of inhaled insulin: from oral cavity to the bloodstream via the lungs Credit: [23]

After inhalation, insulin is propelled through the bronchial pathways through numerous cilia [23]. On reaching the alveoli, it traverses the alveolar wall, undergoes quick absorption into the bloodstream, because the alveolar epithelial lining is only 1-2 µm away from the pulmonary capillary lumen, and then circulates throughout the bloodstream (Figure 2) [18,19,23]. Hence, the pulmonary route of insulin administration offers a quicker onset of response than the subcutaneous route [19]. 

Methods of Delivering Inhaled Insulins

The pulmonary route of insulin has been evaluated via liquid nebulizers, dry powder inhalers, soft mist inhalers, liquid aerosols in cartridge-shaped inhalers, passive inhalers, microprocessor-controlled inhalers, and meter-dose inhalers [18]. Inhalers can be safely and easily used by patients of all age groups, including paediatric and elderly, as well as by patients struggling with various comorbidities [20].

Powdered aerosols have higher pure drug content than liquid aerosols (95% vs. 1%-2%; 98% is water). As a result, a single inhalation of powder aerosols can deliver five times the amount of medication delivered through liquid or nebulizer systems [18].

History of the development of inhaled insulin

**Figure 3:**
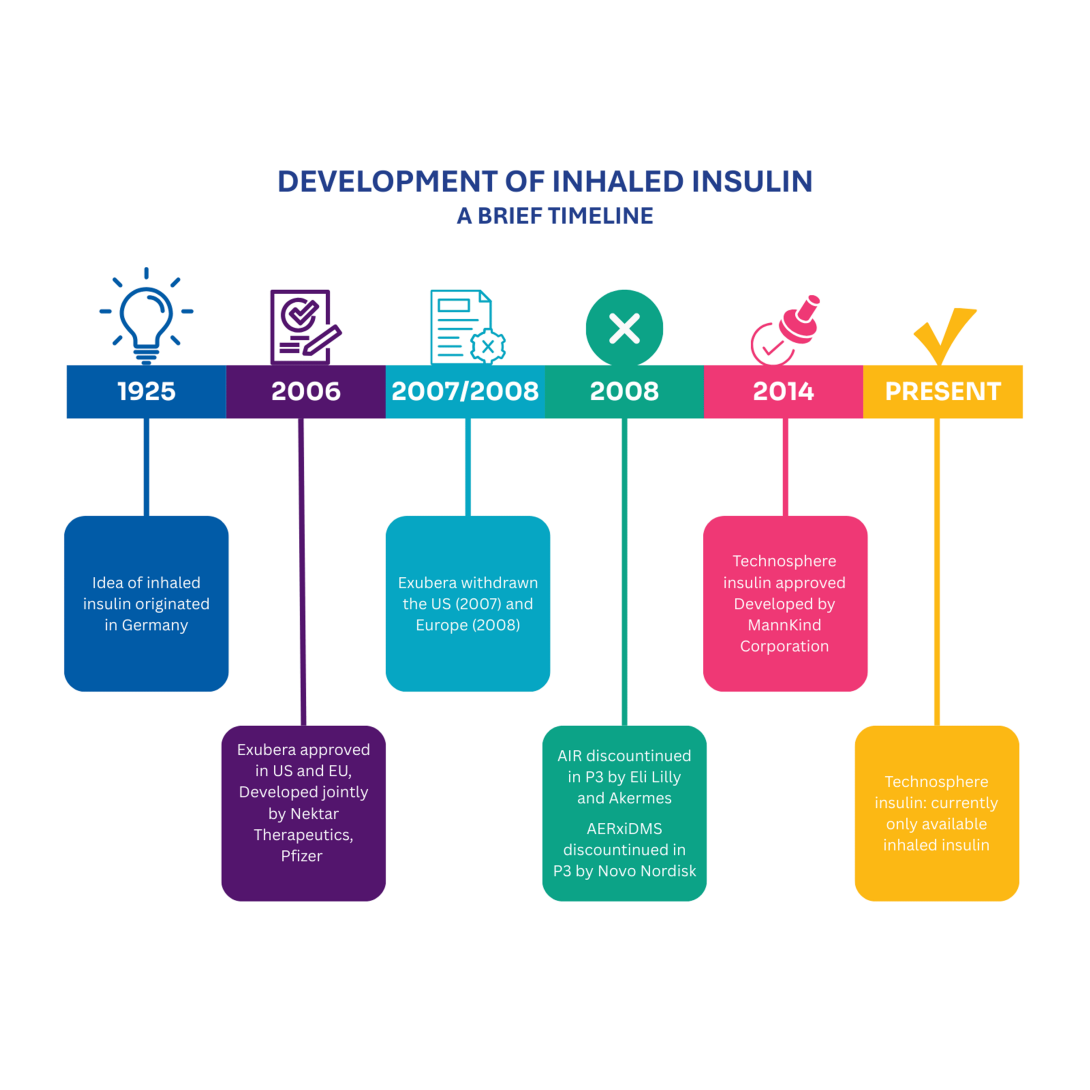
Timeline of inhaled insulin development

The timeline of the inhaled insulin development program is shown in Figure 3.

The first idea of using the pulmonary route for delivering insulin originated in Germany in 1925 [9]. The various types of inhaled insulins developed to date have been compared in Table 2.

**Table 2:** Comparing various inhaled insulins Abbreviations: AERxiDMS, AERx Insulin Diabetes Management System; CDSCO, Central Drugs Standard Control Organization; FDA, Food and Drug Administration; EMA, European Medicines Agency

Sl. No.	Device/Developing Company	Available Form	Regulatory Status	Current Status
1	Exubera/Nektar and Pfizer	Insulin powder in prepackaged blister	FDA and EMA approved [9]	Withdrawn US (2007), Europe (2008) [3,9]
2	AERxiDMS/Novo Nordisk and Aradigm	Pre-prepared insulin liquid blisters through an electronic device	Phase III trials	Development halted in phase III [24,25]
3	AIR/Eli Lilly and Akermes	Dry insulin powder through the mechanical insulin inhaler system	Phase III trials	
4	Afrezza®/Mankind	Dry powder recombinant human insulin in a pre-filled cartridge through a dry powder inhaler system	FDA-approved, CDSCO approved [12,26]	Approved and available in the U.S. and India [12,26]

Exubera® was among the first inhaled insulins to show positive evidence of the efficacy and safety of the pulmonary route of insulin administration [3,15]. It was approved in 2006 for use in adult patients with DM (both type 1 and 2) in the U.S. and Europe [9]. However, Exubera® was discontinued within a year in the U.S. (2007) and Europe (2008) as it was not accepted by both physicians and patients [3,9]. The failure of Exubera® was ascribed to several reasons. The Exubera® device did not provide the discretion that was desired by PwD, as it was bulky and had to be assembled before use. Administering insulin from the device was also more time-consuming when compared to using insulin injections. Patients found it difficult to integrate it into their everyday routine; thus, emphasizing the fact that the success of any non-invasive route of insulin administration was not dependent only on clinical efficacy and safety but also on the availability of a user-friendly device [27].

The AERx® iDMS insulin and the AIR® insulin were the other inhaled insulin types being developed around that time. AERx® iDMS insulin was provided as a pre-packaged blister liquid and administered through an electronic device [9]. This method produced a reproducible inhalation pattern that helped reduce dose frequency and helped healthcare professionals (HCPs) monitor patient adherence. AERx® iDMS increased serum insulin concentrations compared to the subcutaneous route in patients with T1D, showed variability of pharmacodynamic parameters similar to those after administration of subcutaneous insulin, and had great potential as a non-injectable insulin delivery method [9]. The AIR® insulin system is comprised of an insulin inhaler with dry insulin powder. The particle size, with a geometric diameter of 5 to 30 μm, was suitable for effective respiratory airway delivery and distribution [9]. Even though no safety concerns were observed in the clinical trials of AIR® and AERx® iDMS, Eli Lilly and Novo Nordisk, respectively, decided to discontinue their development in 2008 after evaluating commercial and clinical potential compared to other antihyperglycemic therapies and because of the uncertain regulatory environment [24,25].

In this phase of uncertainty, MannKind Corporation continued its development of inhaled insulin. The earliest proof of concept was published in 2000, and finally, in 2014, the US FDA approved Technosphere® insulin (Afrezza®) for adult PwD [3,13]. Currently, Afrezza® is the only available inhaled insulin in the USA and has been in use for more than a decade. In 2018, Cipla Ltd. entered into an agreement with MannKind Corporation, which was followed by a phase III trial conducted for regulatory approval in India [28]. Recently, Afrezza® received Central Drugs Standard Control Organization (CDSCO) approval in India [26].

Technosphere® insulin (Afrezza®)

Afrezza® is a recombinant human insulin, available as a dry powdered formulation adsorbed onto fumaryl diketopiperazine (FDKP) (Technosphere® insulin) that is delivered deep into the lung through the breath-powered Gen2 inhaler [8,29,30]. FDKP is chemically inert and, in a mild acidic environment, self-assembles into microspheres (~2-3 µm) [30-32]. This size range provides a near-optimal aerodynamic diameter for effective delivery to the deep lung [30]. During the precipitation process, peptides such as insulin that are present in the solution get adsorbed onto FDKP at a pH of around 4.5 [31]. These precipitates are freeze-dried and later become available as dry powder for oral inhalation. The FDKP-insulin inhalation powder allows high-dose delivery deep into the lungs with minimal toxicity [31]. FDKP has the ability to dissolve in neutral or alkaline mediums, and hence dissolves rapidly in the alveolar fluid of the deep lung [30,31].

Mechanism of Afrezza® Insulin Delivery

**Figure 4:**
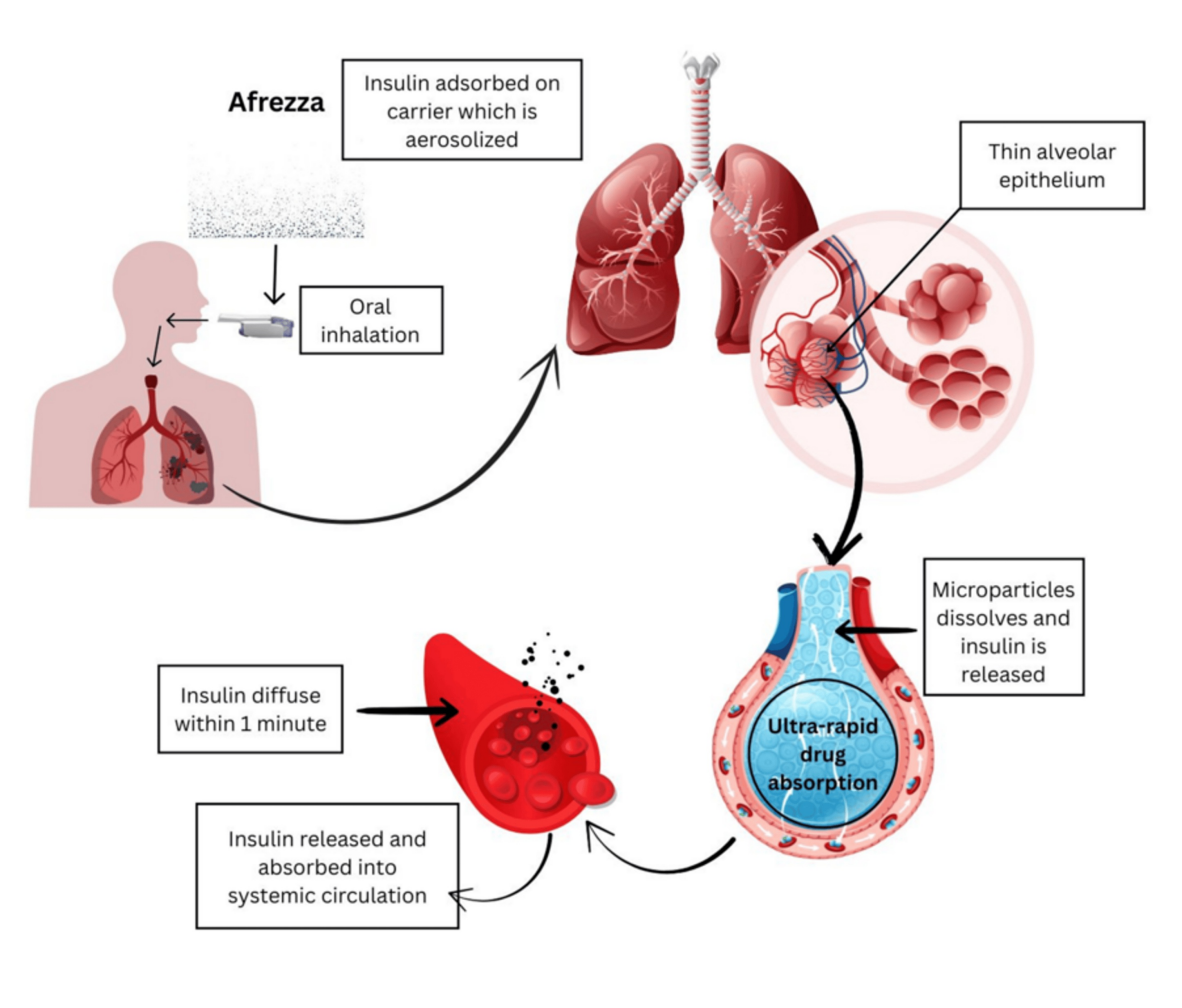
Mechanism of Afrezza® insulin delivery through the pulmonary route Credit: [8,29,33]

On inhalation, Afrezza® is aerosolized, and once it reaches deep into the lungs, FDKP microparticles dissolve at neutral pH, after which insulin is rapidly absorbed and released into the systemic circulation. The mechanism of Afrezza® delivery is shown in Figure 4. The rapid absorption of insulin is believed to be associated with the rapid dissolution of the microparticles [32]. Thereafter, the metabolism and elimination of inhaled insulin are similar to those of regular human insulin. The absorbed FDKP is excreted intact through the kidneys [29,32].

Comparative Effectiveness of Technosphere® Insulin Versus Subcutaneous Insulin

Technosphere® insulin is designed to closely replicate the body’s natural insulin release, making it a suitable option for PwD [34]. The prandial coverage makes it a suitable alternative to bolus insulins [29]. Technosphere® insulin can reduce the four-hour postprandial glucose area under the curve (PPG AUC) by 52% compared to pre-meal human regular insulin. Technosphere® can be used as a prandial insulin support in T2D patients, as it augments PPG and HbA1c reduction in oral antidiabetic drug-treated insulin-naïve patients, and also in patients treated with insulin along with oral antidiabetic drugs (Table 3) [35-37].

**Table 3:** Efficacy and safety of Afrezza® in T1D and T2D Abbreviations: OAD, oral antidiabetic agents; T1D, type 1 diabetes; T2D, type 2 diabetes; TI, technosphere insulin; TP, technosphere placebo

Study	Comparator	Efficacy	Safety
AFFINITY-1 (T1D) [36]	TI + Basal insulin vs Insulin aspart + Basal insulin	Similar HbA1c reduction in the TI arm compared with the insulin aspart arm, -0.21% vs -0.40%; difference between the two arms, 0.19% (within the non-inferiority margin of < 0.4%)	Hypoglycemia: 9.8 vs 14 events/patient/month in the TI vs aspart arm, p < 0.0001; cough incidence: 31.6% vs 2.3% in the TI vs aspart arm
AFFINITY-2 (T2D) [35]	TI + OADs vs Technosphere placebo + OADs	Significant HbA1c reduction, -0.82% vs -0.42% in the TI vs TP arm; difference in HbA1c, -0.4% (p < 0.001)	Hypoglycemia: 1.16 vs 0.5 events/patient/month in the TI vs TP arm, p < 0.0001; cough incidence: 23.7% vs 19.9% in the TI vs TP arm
Phase 3, T2D, India [37]	TI + OADs vs TP + OADs	Significant HbA1c reduction, -0.62% vs -0.20% in the TI vs TP arm; difference in HbA1c, -0.42% (p < 0.0124)	Hypoglycemia incidence: 25.6% vs 16.9% in the TI vs TP arm; cough incidence: 9.0% vs 8.4% in the TI vs Technosphere placebo arm

Afrezza® Patient Acceptability

Perception of insulin therapy was substantially positive post-treatment with Afrezza® [29,34]. An internet survey in adults with T2D showed that patients expressed interest in inhaled insulin mainly because of the benefits of avoiding postprandial hyperglycemia and discomfort or inconvenience [38]. Patient-reported outcomes among individuals with T1D using Technosphere insulin showed greater treatment satisfaction and more positive attitudes toward insulin therapy compared with those using insulin aspart [30].

Afrezza® Safety

Impact of Technosphere® insulin (Afrezza®) on pulmonary function was evaluated in a long-term study. In this 2-year study, it was observed that treatment with Technosphere® insulin resulted in a small, early decrease in forced expiratory volume in 1 second (FEV1), which remained nonprogressive up to 2 years and resolved when treatment was discontinued [30]. The pharmacokinetic/pharmacodynamic properties of Afrezza® are not affected by active and symptomatic upper respiratory tract infection (URTI) [40].

The risk of lung cancer has been raised as a safety concern to the use of inhaled insulin. Two cases of lung malignancies were reported in 3283 patients receiving Technosphere® insulin and no cases reported in 2205 patients receiving active-comparator treatment. Both cases had a history of cigarette exposure (~40 pack-years). Additionally, two cases of non-small-cell lung cancer were diagnosed more than two years after completion of the clinical trial. Thus, these data are insufficient to determine whether Technosphere® insulin has an effect on lung or respiratory tract tumors [39]. Technosphere® insulin is contraindicated in patients with chronic lung disease, as well as in those who smoke or have recently stopped smoking (<6 months) [13,40].

Future directions

Newer inhaled insulins and pulmonary delivery technologies are being investigated. Dance 501, a breath-actuated inhaler, is an insulin-specific vibrating mesh nebulizer (VMN). It can nebulize small insulin doses ranging from 0.050 mL to 0.225 mL [9,41]. The SAMBA-03 and SAMBA-04 trials in T1D and T2D showed a good response compared to insulin Lispro [42]. However, at least 10 breaths, with breath holding, are required to deliver 225 μL of insulin with the Dance-501 VMN [41,43]. Hence, this was not a patient-friendly device [9]. Therefore, the Dance-501 VMN technology is being investigated to improve its design and user-friendliness [41,43].

Since Afrezza® is a new insulin delivery system, not conventionally used, its efficient use can be achieved through patient education, support, and training regarding correct inhalation techniques, glucose monitoring, and dose titrations. Creating awareness, dispelling myths, and addressing queries that either patients or HCPs may have regarding efficacy, safety, or product usage, in a timely and appropriate manner, would be necessary for the successful integration of inhaled insulin into the diabetes management regimen [44].

Any new route of insulin administration would require extensive evidence on efficacy, safety, treatment acceptability, and adherence data. Well-designed studies in different patient populations, and against commonly used subcutaneous insulins, should continue to be conducted. Currently, Afrezza® has been investigated in the INHALE-1 [44,45] and is being investigated in INHALE-GDM [46] trials for the pediatric population (≥4 and <18 years of age), and for pregnant women with gestational diabetes mellitus, respectively. Real-world studies would further provide insights into efficacy, safety, and adherence.

Studies have shown that glycemic control results in improvement in micro- and macrovascular complications. Insulin is among the most efficacious agents known for glucose control. However, studies suggest that insulin initiation is delayed by as much as 11 years after diagnosis of diabetes [47]. The major reasons responsible for this are fear of injections, fear of pain during injections, fear of hypoglycemia, social stigma, and lack of education. Thus, the availability of a non-invasive method of insulin administration, such as inhaled insulin, might help to overcome the multiple barriers associated with insulin therapy and facilitate optimal insulin initiation.

## Conclusions

The review highlights the journey of inhaled insulins and shows their importance in the treatment paradigm of diabetes management. Currently, Afrezza® is the only US FDA-approved, non-invasive insulin. It has several advantages over previously investigated inhaled insulins, such as a small, sleek delivery system, ease of use, dosing in units, quick absorption, and close mimicking of the human body's physiological insulin release. The quest for insulins with improved pharmacokinetics and pharmacodynamics, to mimic physiological insulin, and with a delivery route that makes administration easy and prick-free has been ongoing for more than a century. The evidence with Afrezza® indicates that it meets the key requirements of an ideal prandial insulin, making it a valuable addition to the therapeutic arsenal for diabetes management.
